# A computational model to predict the Diels–Alder reactivity of aryl/alkyl-substituted tetrazines

**DOI:** 10.1007/s00706-017-2110-x

**Published:** 2017-11-29

**Authors:** Dennis Svatunek, Christoph Denk, Hannes Mikula

**Affiliations:** Institute of Applied Synthetic Chemistry, TU Wien, Vienna, Austria

**Keywords:** Cycloadditions, Computational chemistry, Click chemistry, Bioorthogonal chemistry

## Abstract

**Abstract:**

The tetrazine ligation is one of the fastest bioorthogonal ligations and plays a pivotal role in time-critical in vitro and in vivo applications. However, prediction of the reactivity of tetrazines in inverse electron demand Diels–Alder-initiated ligation reactions is not straight-forward. Commonly used tools such as frontier molecular orbital theory only give qualitative and often even wrong results. Applying density functional theory, we have been able to develop a simple computational method for the prediction of the reactivity of aryl/alkyl-substituted tetrazines in inverse electron demand Diels–Alder reactions.

**Graphical Abstract:**

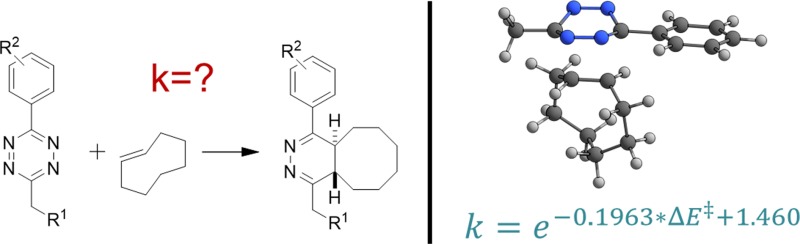

**Electronic supplementary material:**

The online version of this article (10.1007/s00706-017-2110-x) contains supplementary material, which is available to authorized users.

## Introduction

**Fig. 1:**
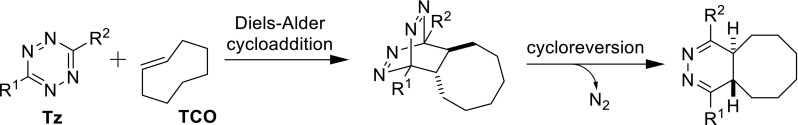
Mechanism of the bioorthogonal ligation of 1,2,4,5-tetrazine (Tz) and *trans*-cyclooctene (TCO)

Tetrazine ligations (TLs) are bioorthogonal inverse electron demand Diels–Alder (IEDDA) initiated cycloadditions proceeding with exceptional high second-order rates of up to 3,300,000 M^−1^ s^−1^ [1]. In TLs, an 1,2,4,5-tetrazine (Tz) reacts with an electron-rich dienophile in an IEDDA reaction followed by cycloreversion under the loss of nitrogen (Fig. 1). Strained alkenes such as norbornenes [2, 3], cyclopropenes [4, 5], and *trans*-cyclooctenes (TCOs) [1, 6–8] are commonly used dienophiles, with TCOs providing the highest reactivity. The rate-determining step is the Diels–Alder cycloaddition, while the cycloreversion has only a low energy barrier and is suspected to show strong non-statistical effects [9].

Due to the high reaction rates, these ligations can be used in time-critical applications such as rapid radiolabeling and pretargeted PET imaging [10–16] and provide high yields within short reaction times even at low concentrations as usually encountered in radiochemistry and in vivo. Therefore, kinetics is one of the most important characteristics of bioorthogonal reactions. However, prediction of reactivities using the chemist’s understanding of organic chemistry, especially of IEDDA reactions, might lead to wrong predictions [17] and only qualitative estimates. In addition, synthesis of tetrazines is often low yielding and involves handling or even requires the production of anhydrous hydrazine (not commercially available in Europe), which limits the feasibility of screening for high Diels–Alder reactivity. Hence, there is the need of reliable computational tools to predict the reactivity of various tetrazines in TLs.

Herein, we introduce a computational model for the prediction of the reactivity of aryl/alkyl-substituted Tz in the cycloaddition with *trans*-cyclooctene (TCO), thus eliminating the need for expensive and even dangerous synthetic work, finally enabling in silico screening for tetrazines with desired reactivity.

While 3,6-bisaryl- and 3-aryl-substituted Tz show the highest reactivity, aryl/alkyl-substituted Tz are commonly used due to higher stability [18, 19] and show favorable properties in Tz-triggered bioorthogonal elimination reactions [20, 21].

## Results and discussion

**Fig. 2:**
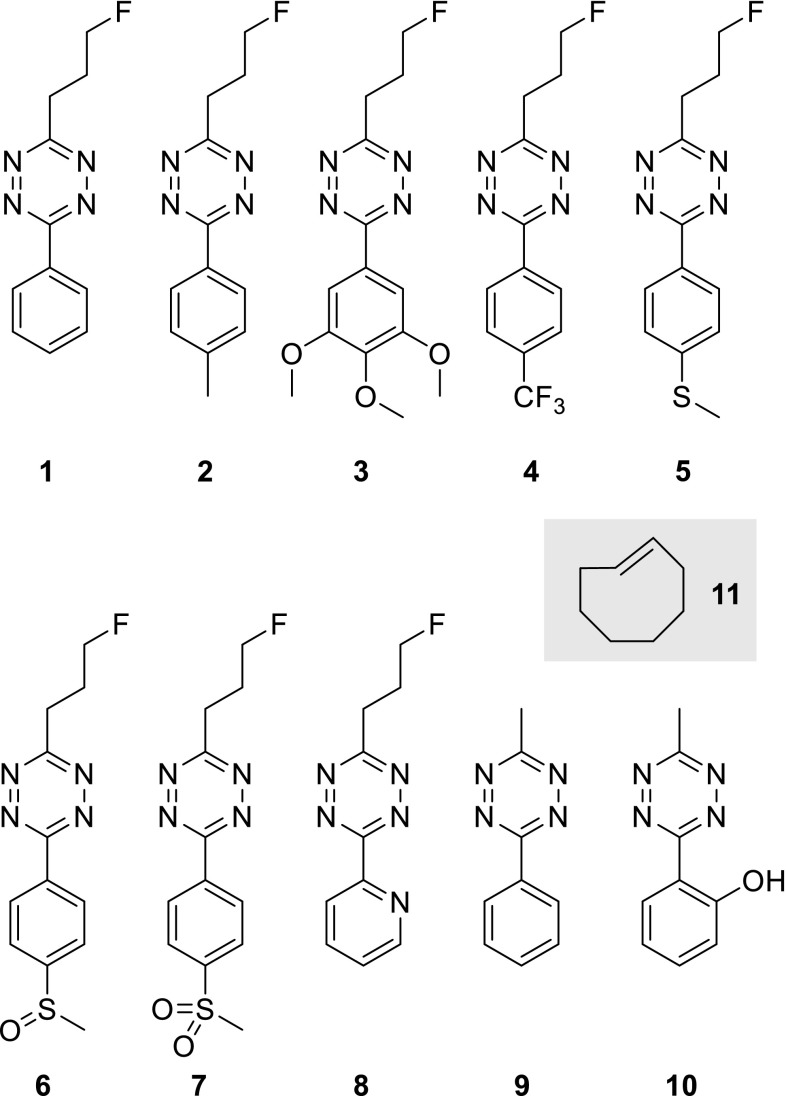
Investigated aryl/alkyl substituted 1,2,4,5-tetrazines in the IEDDA reaction with *trans*-cyclooctene (**11**)

Recently, we have investigated the reactivity of several 3-aryl-6-(3-fluoropropyl)-1,2,4,5-tetrazines **1**–**8** as chemical probes for rapid radiolabeling and pretargeted PET imaging (Fig. 2). While the alkyl substituent is the same for all eight tetrazines the aryl component shows considerable variation including electron-rich and electron-poor aryl groups. In addition, Tz **9** and **10** were included to investigate the influence of the alkyl group and an *ortho*-substituted aryl residue, respectively.

**Fig. 3:**
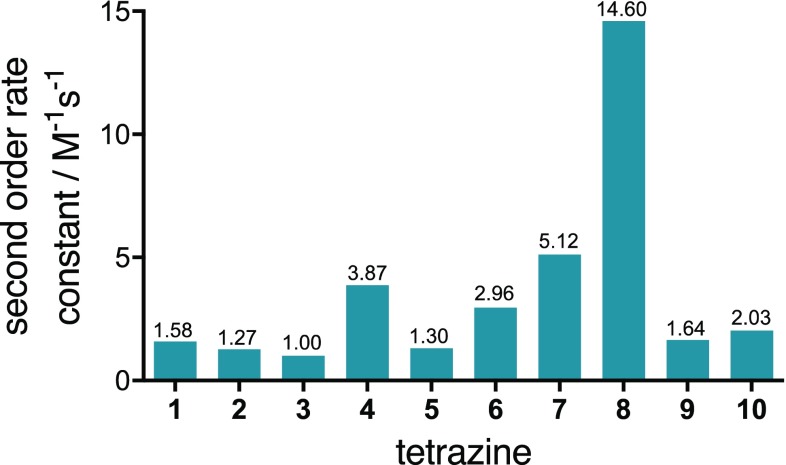
Second-order rate constants of tetrazines **1**–**10** with *trans*-cyclooctene (**11**) in 1,4-dioxane at 25 °C

The second-order rate constants of Tz **1**–**10** in the reaction with *trans*-cyclooctene **11** at 25 °C in anhydrous 1,4-dioxane were measured by stopped-flow spectrophotometry, which gave rates ranging from 1.00 M^−1^ s^−1^ for electron-rich trimethoxyphenyl-substituted Tz **3** to 14.6 M^−1^ s^−1^ for Tz **8** bearing an electron withdrawing 2-pyridyl substituent (Fig. 3).

These experimental results were selected as a basis for the construction of a predictive computational tool. DFT was successfully used in the past by our group [16, 22] and others [7, 17, 23, 24] to predict or explain the reactivity of dienophiles and tetrazines in the tetrazine ligation. Therefore, the Minnesota density functional M06-2X in combination with the 6-311+G(d,p) basis set, was used as model chemistry. This density functional has been proven to produce accurate results for thermodynamics of cycloaddition reactions [25, 26].

**Fig. 4:**
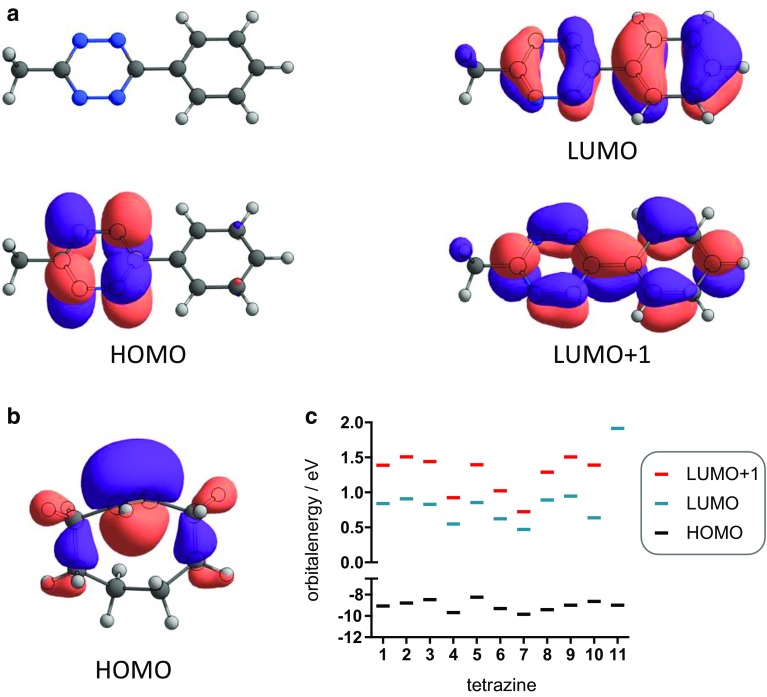
a HOMO, LUMO, and LUMO+1 of 3-methyl-6-phenyl-Tz (**9**); b HOMO of TCO (**11**); c energy levels of selected orbitals for tetrazines **1**–**10** and TCO (**11**)

Diels–Alder reactions can be described by HOMO/LUMO interactions using the frontier molecular orbital (FMO) theory. In case of the IEDDA cycloaddition the main orbital interaction is between a low-lying unoccupied orbital of the dienophile, usually being the LUMO+1 for aryl/alkyl tetrazines (Fig. 4a) [17], and the HOMO of the electron-rich dienophile (in this case TCO, Fig. 4b).

**Fig. 5:**
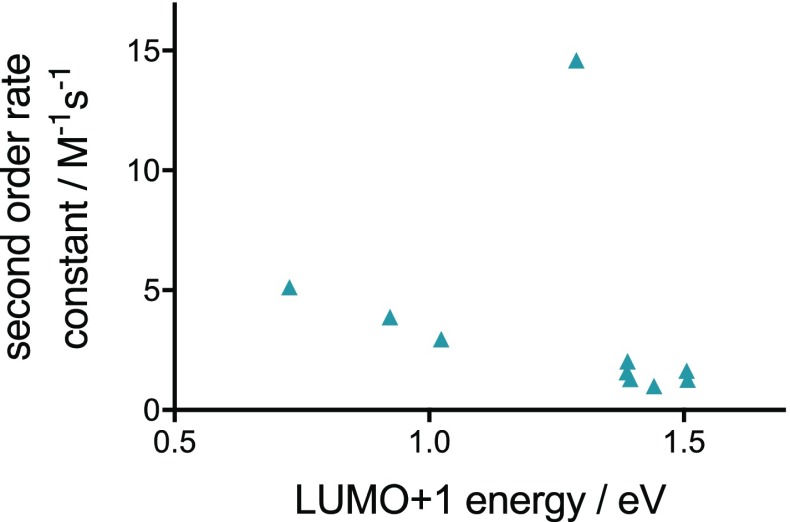
Second-order rate constants plotted against HF/6-311+G(d,p)//M06-2X/6-311+G(d,p) calculated LUMO+1 energies of the corresponding tetrazine

According to the FMO theory, a smaller energy gap between the interacting orbitals facilitates the reaction. Thus, one might expect that a more electron withdrawing and thus LUMO+1-lowering substituent accelerates the reaction, while an electron-rich aryl substituent will decrease reactivity. HF/6-311+G(d,p)//M06-2X/6-311+G(d,p)-calculated orbital energies are shown in Fig. 4c. Tz **4** and **7** bearing an electron-withdrawing trifluoromethyl or sulfone group, respectively, show the lowest LUMO and LUMO+1 energies. However, the tetrazine with the highest reactivity, Tz **8**, has one of the highest LUMO and a rather high LUMO+1 energy within the series. As shown in Fig. 5, there is no significant correlation between the LUMO+1-energy levels and the rate constants (*R* ^2^ = 0.07). This can be rationalized by the fact that FMO interactions are not the only major contributors to activation energies in such cycloadditions [17, 27–29]. Therefore, the FMO theory cannot be applied for the reliable prediction of the Diels–Alder reactivity of aryl/alkyl-substituted Tz.

**Fig. 6:**
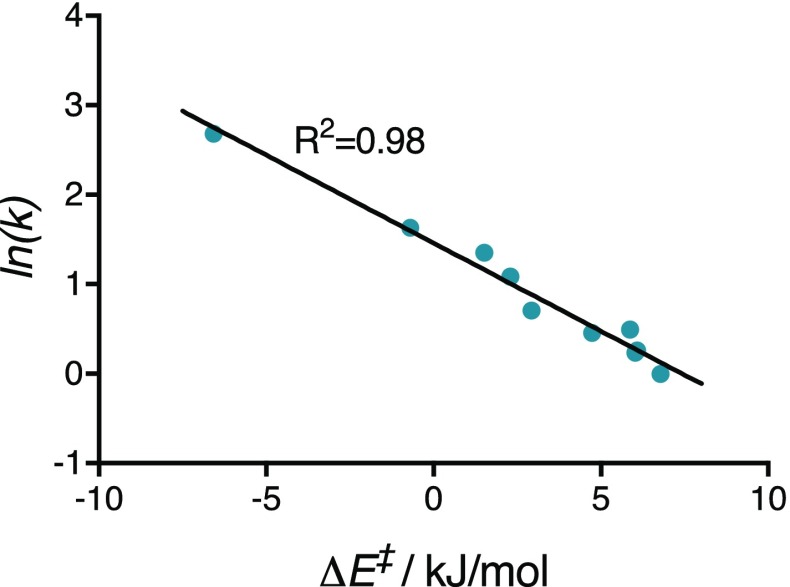
Correlation between natural logarithm of second-order rate constants and M06-2X/6-311+G(d,p) calculated energies of activation

However, the calculated energies of activation (Δ*E* ^‡^) of the reactions between tetrazines **1**–**10** and TCO (**11**) show excellent linear correlation with the natural logarithm of the measured rate constants, as per transition state theory (Fig. 6). M06-2X/6-311+G(d,p) calculated energies of activation can, therefore, be used to reliably predict the reactivity of aryl/alkyl-Tz in IEDDA reactions with *trans*-cyclooctenes.

Using the linear correlation between ln(*k*) and calculated Δ*E* ^‡^, Eq. (1) can be constructed which allows the prediction of the rate constant of new aryl/alkyl-Tz for the reaction with TCO (**11**) in anhydrous 1,4-dioxane at 25 °C.

1 $$k = {\text{e}}^{{ - 0.1963*\Delta E^{\ddag } + 1.460}}$$

## Conclusion

Prediction of the reactivity of Tz in bioorthogonal ligation reactions is essential to reduce synthetic work, the resulting costs and associated risks. However, the chemist’s prediction of these reactivities, mainly based on frontier molecular orbital theory, can only yield qualitative or even wrong results as shown in this work. We were able to develop a computational method for the prediction of reactivities of aryl/alkyl-substituted 1,2,4,5-tetrazines in the reaction with *trans*-cyclooctene based on M06-2X-calculated energies of activation. This method is computationally cheap as it requires only the optimization of the tetrazine and the corresponding transition state with *trans*-cyclooctene at the M06-2X/6-311+G(d,p) level of theory, which can even be done on an average desktop PC within hours.

We are convinced that our method will aid the development of new aryl/alkyl tetrazines for bioorthogonal applications, and represents a step forward to the development of a universal computational tool being able to predict the reactivity of tetrazines with various substitution patterns.

## Experimental

### Computational methods

Calculations were performed at the M06-2X/6-311+G(d,p) level of theory in the gas phase using the Gaussian 09 Rev. D.01 software package [30]. Vibrational analysis was performed to confirm stationary points are energetic minima or transition states, respectively. Orbital energies were calculated by performing a HF/6-311+G(d,p) single-point calculation on M06-2X/6-311+G(d,p) optimized structures. Δ*E* ^‡^ was determined by calculating the difference in energies between transition state and reactants. Data analysis was preformed using Gaussview 5 and Chemcraft. XYZ coordinate files of all reactants and transition states are available as electronic supplementary material. Calculated energies for Tz **1**–**10** and the respective transition states (for the reaction with TCO) are shown in Table 1.

**Table 1:** Calculated total electronic energies of compounds **1**–**11** and transition states for the reaction between **1**–**10** and **11**, and calculated energies of activation (Δ*E* ^‡^)

Compound	*E*(Tz)/hartree	*E*(TS)/hartree	Δ*E* ^‡^/kJ mol^−1^
**1**	− 744.464487	− 1057.63536	4.74
**2**	− 783.771996	− 1096.942379	6.02
**3**	− 1087.994366	− 1401.164459	6.78
**4**	− 1081.515706	− 1394.687804	1.52
**5**	− 1181.953194	− 1495.123556	6.08
**6**	− 1257.12132	− 1570.293123	2.29
**7**	− 1332.328085	− 1645.501028	− 0.70
**8**	− 760.498963	− 1073.674144	− 6.57
**9**	− 566.619295	− 879.789737	5.87
**10**	− 641.851471	− 955.023032	2.93
TCO (**11**)	− 313.172677	–	–

### Synthesis

*Trans*-cyclooctene (**11**) and tetrazines **9** and **10** were prepared following known procedures [31, 32]. The synthesis of compounds **1**–**8** will be published elsewhere (manuscript in preparation).

### Kinetic measurements

Kinetic measurements were performed on a SX20 stopped-flow spectrophotometer (Applied Photophysics, UK) using a 535 nm LED light source. A 4 mM solution of **11** and approximately 0.1 mM solutions of Tz **1**–**10** were prepared in anhydrous 1,4-dioxane (note: for correct measurements it is of utmost importance to use anhydrous 1,4-dioxane, since even small amounts of water will accelerate the reaction, leading to irreproducible data). These solutions were loaded into the driver syringes and equal volumes of TCO and Tz solution were mixed, resulting in concentrations of **2** and 0.05 mM, respectively. The reaction progress was followed by measuring the absorption around 535 nm. All measurements were performed in triplicates. Observed reaction constants (*k*_obs_) were determined by non-linear regression (one-phase-decay) using Prism 6 (Graphpad) and second-order rate constants (*k*) were calculated from *k*_obs_. Table 2 lists *k*_obs_, *c*_TCO_, *c*_Tz_ and *k* for all reactions.

**Table 2:** Observed rate constants, concentrations of TCO (**11**) and the corresponding Tz, and calculated second-order rate constants of the IEDDA reaction between Tz **1**–**10** and **11**

Reaction	*k*_obs_/s^−1^	*c*_TCO_/M	*c*_tetrazine_/M	*k*/M^−1^ s^−1^
**1** + **11**	0.003278	0.002081	0.000064	1.58
**2** + **11**	0.002652	0.002096	0.00005	1.27
**3** + **11**	0.00205	0.002051	0.000049	1.00
**4** + **11**	0.007801	0.002015	0.00005	3.87
**5** + **11**	0.002703	0.002081	0.000034	1.30
**6** + **11**	0.005811	0.00196	0.000046	2.96
**7** + **11**	0.009894	0.001933	0.000103	5.12
**8** + **11**	0.03064	0.002096	0.00005	14.6
**9** + **11**	0.00461	0.002813	0.000058	1.64
**10** + **11**	0.004514	0.002224	0.000061	2.03

## Electronic supplementary material

Below is the link to the electronic supplementary material.

Supplementary material 1 (XYZ 2 kb)

Supplementary material 2 (XYZ 3 kb)

Supplementary material 3 (XYZ 2 kb)

Supplementary material 4 (XYZ 3 kb)

Supplementary material 5 (XYZ 3 kb)

Supplementary material 6 (XYZ 4 kb)

Supplementary material 7 (XYZ 2 kb)

Supplementary material 8 (XYZ 3 kb)

Supplementary material 9 (XYZ 2 kb)

Supplementary material 10 (XYZ 3 kb)

Supplementary material 11 (XYZ 2 kb)

Supplementary material 12 (XYZ 3 kb)

Supplementary material 13 (XYZ 2 kb)

Supplementary material 14 (XYZ 4 kb)

Supplementary material 15 (XYZ 2 kb)

Supplementary material 16 (XYZ 3 kb)

Supplementary material 17 (XYZ 2 kb)

Supplementary material 18 (XYZ 3 kb)

Supplementary material 19 (XYZ 2 kb)

Supplementary material 20 (XYZ 3 kb)

Supplementary material 21 (XYZ 2 kb)
